# Robust Detection of Impaired Resting State Functional Connectivity Networks in Alzheimer's Disease Using Elastic Net Regularized Regression

**DOI:** 10.3389/fnagi.2016.00318

**Published:** 2017-01-04

**Authors:** Stefan J. Teipel, Michel J. Grothe, Coraline D. Metzger, Timo Grimmer, Christian Sorg, Michael Ewers, Nicolai Franzmeier, Eva Meisenzahl, Stefan Klöppel, Viola Borchardt, Martin Walter, Martin Dyrba

**Affiliations:** ^1^Department of Psychosomatic Medicine, University of RostockRostock, Germany; ^2^German Center for Neurodegenerative Diseases, Site Rostock/GreifswaldRostock, Germany; ^3^Institute of Cognitive Neurology and Dementia Research and Department of Psychiatry and Psychotherapy, Otto von Guericke UniversityMagdeburg, Germany; ^4^German Center for Neurodegenerative Diseases, Site MagdeburgMagdeburg, Germany; ^5^Department of Psychiatry and Psychotherapy, Klinikum rechts der Isar, Technische Universität MünchenMunich, Germany; ^6^Department of Neuroradiology of Klinikum rechts der Isar, Technische Universität MünchenMunich, Germany; ^7^Department of Psychiatry of Klinikum rechts der Isar, Technische Universität MünchenMunich, Germany; ^8^TUM-Neuroimaging Center, Technische Universität MünchenMunich, Germany; ^9^Institute for Stroke and Dementia Research, Klinikum der Universität München, Ludwig-Maximilians-UniversitätMunich, Germany; ^10^Department of Psychiatry, Klinikum der Universität München, Ludwig-Maximilians-UniversitätMunich, Germany; ^11^Department of Psychiatry and Psychotherapy, Section of Gerontopsychiatry and Neuropsychology, Faculty of Medicine, University of FreiburgFreiburg, Germany; ^12^University Hospital of Old Age PsychiatryBern, Switzerland; ^13^Leibniz Institute for NeurobiologyMagdeburg, Germany; ^14^Department of Psychiatry, University of TübingenTübingen, Germany

**Keywords:** regularization, diagnostic imaging, feature selection, functional magnetic resonance imaging (fMRI), Alzheimer's disease

## Abstract

The large number of multicollinear regional features that are provided by resting state (rs) fMRI data requires robust feature selection to uncover consistent networks of functional disconnection in Alzheimer's disease (AD). Here, we compared elastic net regularized and classical stepwise logistic regression in respect to consistency of feature selection and diagnostic accuracy using rs-fMRI data from four centers of the “German resting-state initiative for diagnostic biomarkers” (psymri.org), comprising 53 AD patients and 118 age and sex matched healthy controls. Using all possible pairs of correlations between the time series of rs-fMRI signal from 84 functionally defined brain regions as the initial set of predictor variables, we calculated accuracy of group discrimination and consistency of feature selection with bootstrap cross-validation. Mean areas under the receiver operating characteristic curves as measure of diagnostic accuracy were 0.70 in unregularized and 0.80 in regularized regression. Elastic net regression was insensitive to scanner effects and recovered a consistent network of functional connectivity decline in AD that encompassed parts of the dorsal default mode as well as brain regions involved in attention, executive control, and language processing. Stepwise logistic regression found no consistent network of AD related functional connectivity decline. Regularized regression has high potential to increase diagnostic accuracy and consistency of feature selection from multicollinear functional neuroimaging data in AD. Our findings suggest an extended network of functional alterations in AD, but the diagnostic accuracy of rs-fMRI in this multicenter setting did not reach the benchmark defined for a useful biomarker of AD.

## Introduction

Many studies have identified altered functional connectivity networks in resting state examinations of Alzheimer's disease (AD) patients compared to controls using functional imaging techniques such as FDG-PET or resting state functional MRI (rs-fMRI) (for a recent review see Teipel et al., 2016). Typically AD dementia impairs functional connectivity in the default mode network (DMN; Greicius et al., 2004), but AD pathological changes and ensuing functional disruptions have been shown to extend beyond the regions of the DMN (Agosta et al., 2012; Grothe et al., 2016).

To identify the network characteristics of AD-related changes in functional imaging data, most studies have employed stepwise or multiple linear regression approaches (Agosta et al., 2012; Koch et al., 2012; Sheline and Raichle, 2013). However, features from rs-fMRI and other functional imaging data are often highly collinear across regions, and linear regression approaches are known to be highly sensitive toward collinearity (James et al., 2013; Section 3.3.6). In the presence of a high number of features relative to the number of available observations (Tibshirani, 2011) and when features are collinear (Hoerl and Kennard, 1970; Tibshirani, 1996), regularization techniques have been established for dimension reduction and feature selection. More recently, regularized models, using an elastic net penalty (Zou and Hastie, 2005; Zou and Zhang, 2009), have been applied to multimodal neuroimaging studies to reduce the effect of multicollinearity on feature selection (Trzepacz et al., 2014; Teipel S. J. et al., 2015; Schouten et al., 2016; de Vos et al., 2016).

Here, we used rs-fMRI data from a multicenter study to compare accuracy of group separation, as well as stability of regional feature selection and ensuing identification of cortical networks discriminating AD patients and controls between cross-validated regularized logistic regression with an elastic net penalty and classical stepwise logistic regression. We hypothesized that elastic net logistic regression would lead to more generalizable feature selection and more consistent network identification than classical stepwise logistic regression. Of note, the principles of these methods, except the elastic net penalty, represent textbook knowledge from statistical learning literature, but adoption of these methods to the burning issue of highly collinear features in neuroimaging research is still slow.

## Materials and methods

For the current study, we used data from 53 patients with clinically probable AD according to NINCDS-ADRCA criteria (McKhann et al., 1984) and 118 healthy elderly control individuals that have been retrieved retrospectively from four sites within the framework of the “German resting-state initiative for diagnostic biomarkers” (http://www.psymri.org). Distribution of demographic characteristics of participants across sites is summarized in Table 1.

**Table 1:** **Demographic characteristics**.

	**AD**	**Controls**
No. cases (women)^a^	53 (31)	118 (61)
Age (SD) [years]^b^	72.4 (8.8)	70.4 (6.2)
MMSE (SD), number^c^	22.5 (4.4), 53	28.8 (1.0) 97
MoCA (SD), number	–	26.4 (2.1), 19
Education (SD) [years]^d^	11.4 (2.1)	13.6 (3.1)

*MMSE, Mini Mental State Examination (Folstein et al., 1975); MoCA, Montreal Cognitive Assessment (Nasreddine et al., 2005)*.

a *Not significantly different between groups, χ^2^ = 0.68, 1 df, p = 0.41*.

b *Not significantly different between groups, t = 1.67, 169 df, p = 0.96*.

c *significantly different between groups, Mann-Whitney U-test, p < 0.001*.

d *significantly different between groups, t = −4.72, 168 df, p < 0.001*.

All participants were free of any significant neurological, psychiatric, or medical condition (except for AD in patients), in particular cerebrovascular apoplexy, vascular dementia, depression, or subclinical hypothyroidism, as well as substance abuse. Healthy controls were required to have no cognitive complaints and scored within one standard deviation of the age and education adjusted norm in all subtests of the Consortium to Establish a Registry of Alzheimer's Disease (CERAD) cognitive battery (Morris et al., 1989).

Written informed consent was provided by all subjects, or their representatives. The study was approved by local ethics committees at each of the participating centers, and has been conducted in accord with the Helsinki Declaration of 1975.

### Imaging and data acquisition

The data used in this study were obtained from four different 3.0 Tesla MRI scanners. Acquisition parameters for the rs-fMRI sequences are given in Table 2. In one center (site I), the subjects were instructed to keep their eyes open, whereas in the remaining centers (sites II-IV) all subjects were requested to close their eyes, relax, but not to fall asleep. Functional MRI was based on echo-planar imaging using scan durations between 6 and 8.7 min for the rs-fMRI sequence. The number of acquired time points was between 120 and 200 with a voxel size ranging from 2 × 2 × 2.6 up to 3.28 × 3.28 × 4.4 mm^3^ (Table 2). For anatomical reference, high-resolution T1-weighted gradient echo sequences with an isotropic resolution of 1 mm^3^ were also obtained from all scanners during the same session.

**Table 2:** **Scanner characteristics**.

**Center**	**Model**	**Manufacturer**	**TR [s]**	**TE [s]**	**Volumes**	**Voxel size [mm^3^]**	**Gap [mm]**
I	TrioTim	Siemens	2.61	0.030	200	3 × 3 × 3.6	0.6
II	Verio	Siemens	3	0.030	120	2 × 2 × 2.6	0.6
III	Verio	Siemens	2.58	0.030	180	3.5 × 3.5 × 3.5	0
IV	Trio	Siemens	3	0.030	120	3.28 × 3.28 × 4.4	0.4

### MR processing

Functional MRI data processing was carried out using Data Processing Assistant for Resting-State fMRI (DPARSF 3.2) (Chao-Gan and Yu-Feng, 2010), considering the recommendations from a recent systematic evaluation of processing alternatives (Shirer et al., 2015). After the removal of the first six images to account for gradient field stabilization, the rs-fMRI data was slice time corrected and realigned to the temporal mean image. The anatomical T_1_-weighted image of each participant was coregistered to the mean functional image and subsequently segmented into gray matter, white matter, and cerebrospinal fluid (CSF) partitions using the Voxel-based Morphometry (VBM8) toolbox (Gaser et al., 1999) that extends Statistical Parametric Mapping (SPM8) (Friston et al., 2007). The Diffeomorphic Anatomical Registration Through Exponentiated Lie algebra (DARTEL) algorithm (Ashburner, 2007) was applied to normalize the T_1_-weighted images to the Montreal Neurological Institute (MNI) reference coordinate system using the default brain template included in VBM8. The deformation fields generated by DARTEL were used to project the functional scans from each subjects' native image space into the MNI reference space. We combined this step with the reslicing of all functional data to an isotropic resolution of 3 mm. The subsequent nuisance regression included covariates of head movement (rotation, translation, and first and second order derivatives) and the mean time courses for the global brain signal, the white matter segment signal, and the CSF segment signal. Although global signal regression was found to introduce negative correlations (Murphy et al., 2009; Shirer et al., 2015), studies consistently reported that it effectively increases the signal-to-noise ratio (Yan et al., 2013; Power et al., 2014; Shirer et al., 2015). Recently, Shirer et al. evaluated the influence of global signal regression on group separation but only found a minor, non-significant effect (Shirer et al., 2015). Subsequently, the images were band-pass filtered using the frequency band 0.1–0.01 Hz. For each individual the time series of signal was extracted for each of the 84 functionally defined regions of the Greicius atlas (Shirer et al., 2012). Pearson's correlation coefficients were computed for the 3486 possible pairs of correlations between these 84 regions (Shirer et al., 2012). Finally, Pearson correlation coefficients of the signal time courses were adjusted to be normally distributed using Fisher's Z-transform (Fisher, 1915): z = 0.5 ln [(1+r)/(1–r)].

### Statistical analysis

#### Demographic characteristics

Baseline demographic characteristics were compared between AD and control cases using parametric and non-parametric tests as required: age and years of education were compared between groups using Student's *t*-test, gender distribution using Chi^2^ test, and neuropsychological test results using non-parametric Mann-Whitney *U*-test.

#### Prediction of group membership

We compared two regression models for prediction of group membership (AD vs. controls) in respect to two outcomes, (i) the accuracy of prediction as determined by the area under the receiver operating characteristics curve (AUC), and (ii) the consistency of feature selection.

**Figure 1:**
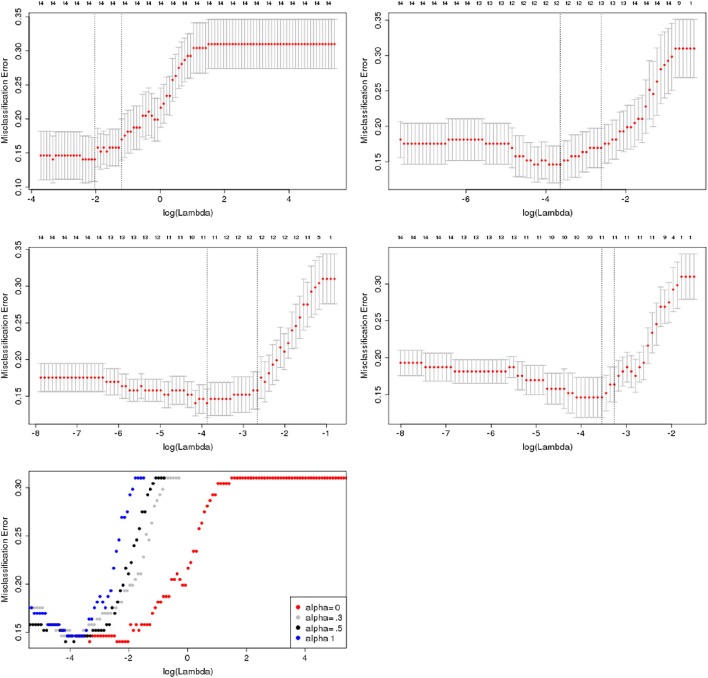
**Selection of alpha parameter for penalized logistic regression**. Misclassification error plotted against the range of lambda values (plotted on a logarithmic scale) for different values of α for a penalized logistic regression on the rs-fMRI data. Numbers on top of each graph indicate the number of selected variables. Error bars indicate the bootstrapped standard deviation for the misclassification error for each lambda value. The left bottom plot shows the different deviance curves on a unified scale, indicating that α = 0.5 yields the lowest deviance together with α = 0, corresponding to a ridge regression model.

The two regression models encompassed:

1. bidirectional (backward and forward) stepwise unpenalized logistic regressions using the function *step* in R (The R Foundation for Statistical Computing). The function weights the choices via the Akaike information criterion (AIC), which takes account of the total number of fitted parameters.
2. penalized logistic regression models with an elastic net penalty, as determined using the R package *glmnet* (available at http://cran.r-project.org/web/packages/glmnet/index.html). Elastic net regression is controlled by two parameters, (i) alpha, which sets the degree of mixing between two types of regularized regression, namely ridge regression (regularization by squared L_2_ norm; alpha = 0) and the Lasso (Least Absolute Shrinkage and Selection Operator, regularization by L_1_ norm; alpha = 1), and (ii) lambda, defining the strength of regularization (Friedman et al., 2010). Alpha was selected to be 0.5, corresponding to a full elastic net penalty, which minimized the partial likelihood deviance of the model (see Figure 1). Lambda was determined using grid search with 100-fold cross-validation. The optimal lambda was determined as the mean across 100 iteratively determined lambda values minimizing the deviance of the model. The optimal lambda value was determined for each bootstrap iteration in the training data and applied to the test data as defined below. Details of this method can be found in the appendix.

Both models were determined using strict cross-validation procedures. Random samples of 2/3 of the data were drawn 1000 times to train the prediction models (training data). For both regression models, the prediction accuracy was determined using the remaining 1/3 as test data. Parameter optimization, i.e., selection of optimal lambda and (stepwise) feature selection, was conducted in the training data and subsequently applied to the test data. Prior to model building, the feature space was restricted through determining the set of variables which correlate with diagnosis with a Pearson's correlation coefficient of |r| > 0.35 in the training data, resulting in an average number of 36 included predictor variables across the bootstrapped repetitions.

In a second analysis, dummy coded center variables were forced as additional variables into the models to determine the effect of center on model accuracy and feature selection.

To check for multicollinearity of the stepwise logistic regression models, we determined the variance inflation factor (VIF) (Belsley, 1991) for each independent variable on the set of the remaining independent variables using the function vif in R package “car” (available at https://cran.r-project.org/web/packages/car/index.html).

## Results

### Demographic characteristics

Demographic characteristics are summarized in Table 1. AD patients and controls were not significantly different in age (*t* = 1.67, 169 df, *p* = 0.96) or sex distribution (Chi^2^ = 0.68, 1 df, *p* = 0.41). Both groups differed significantly in years of education (*t* = −4.72, 168 df, *p* < 0.001), with less years of education in the AD cases, and, as expected, AD patients scored significantly lower than healthy controls in the MMSE score (*p* < 0.001).

### Prediction of group membership

**Figure 2:**
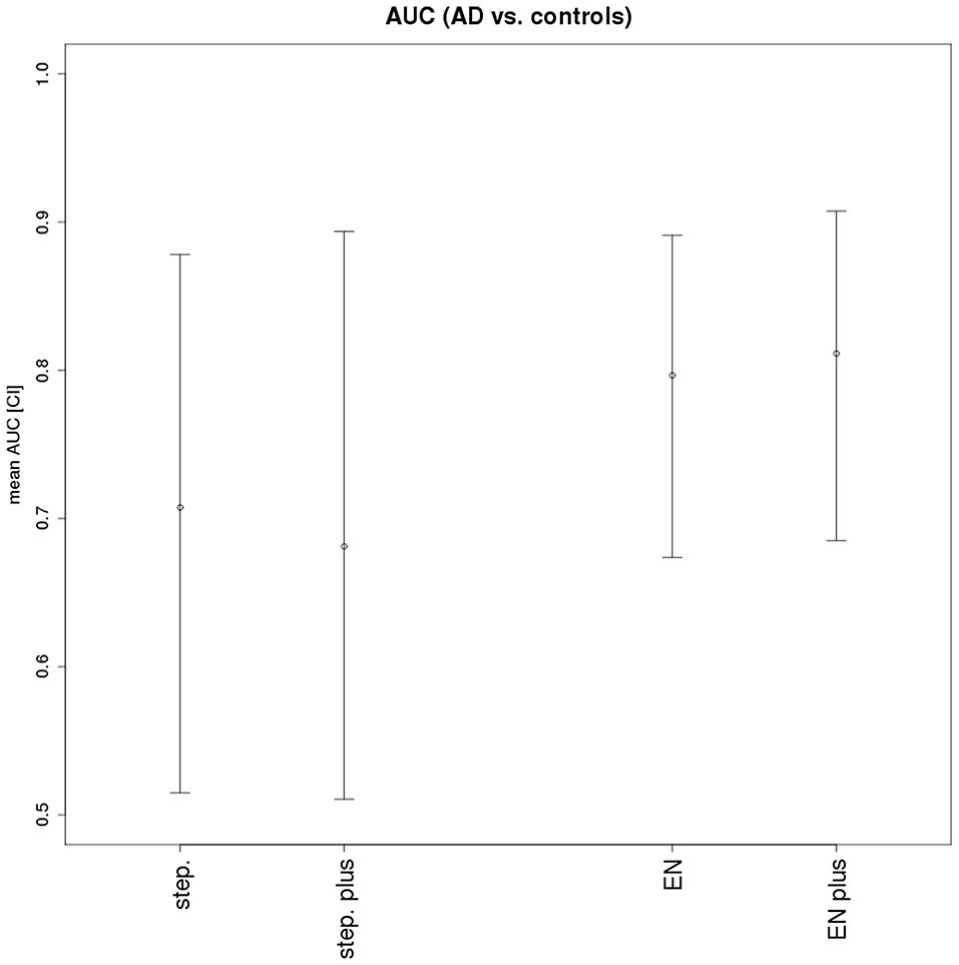
**Areas under the ROC curves for stepwise and elastic net logistic regression**. AUC and 2.5/97.5 percentile confidence intervals for stepwise logistic regression without scanner (step.) and with scanner forced into the model (step. plus), and for elastic net logistic regression without scanner (EN) and with scanner forced into the model (EN plus).

**Figure 3:**
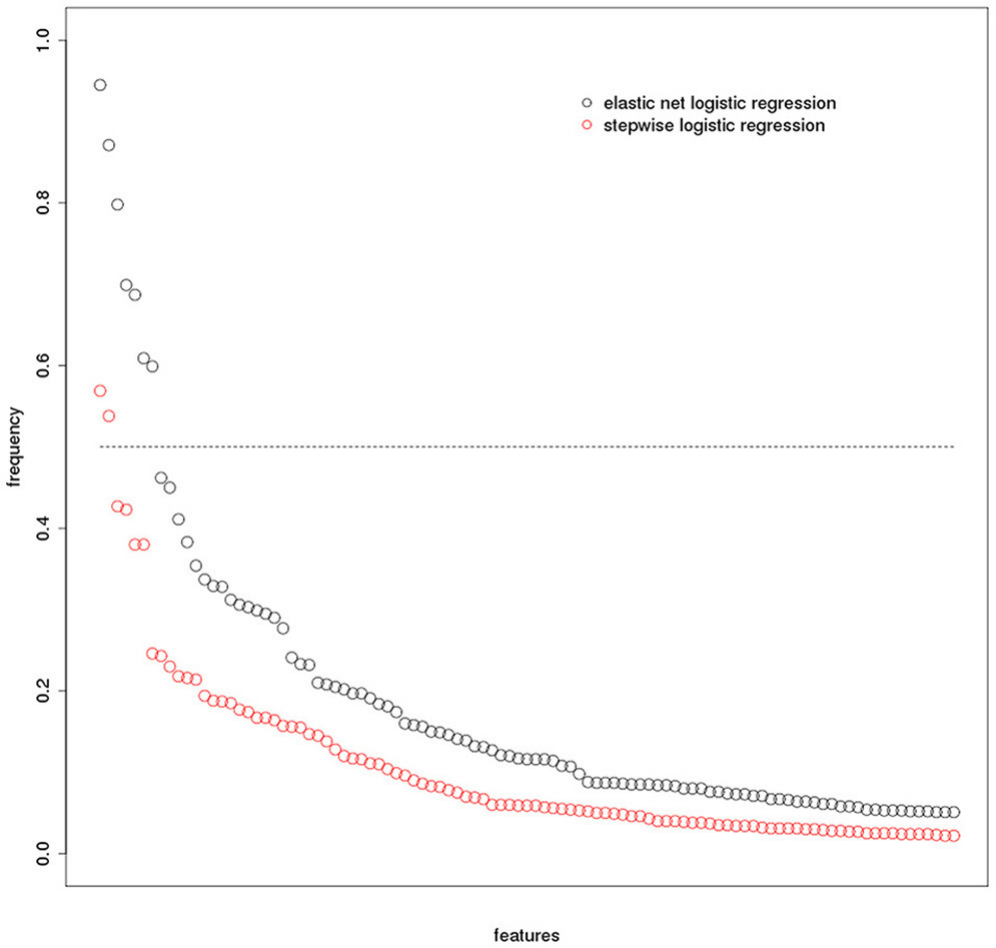
**Feature selection frequency plot**. Frequency of selected features (based on 1000 bootstrap iterations) for elastic net and stepwise logistc regression. Please note that the x-axis represents the features that were sorted according to their frequency independently within each model. Therfore, the same position on the x-axis does not indicate the same feature for the elastic net and the stepwise logistic regression models, respectively.

**Figure 4:**
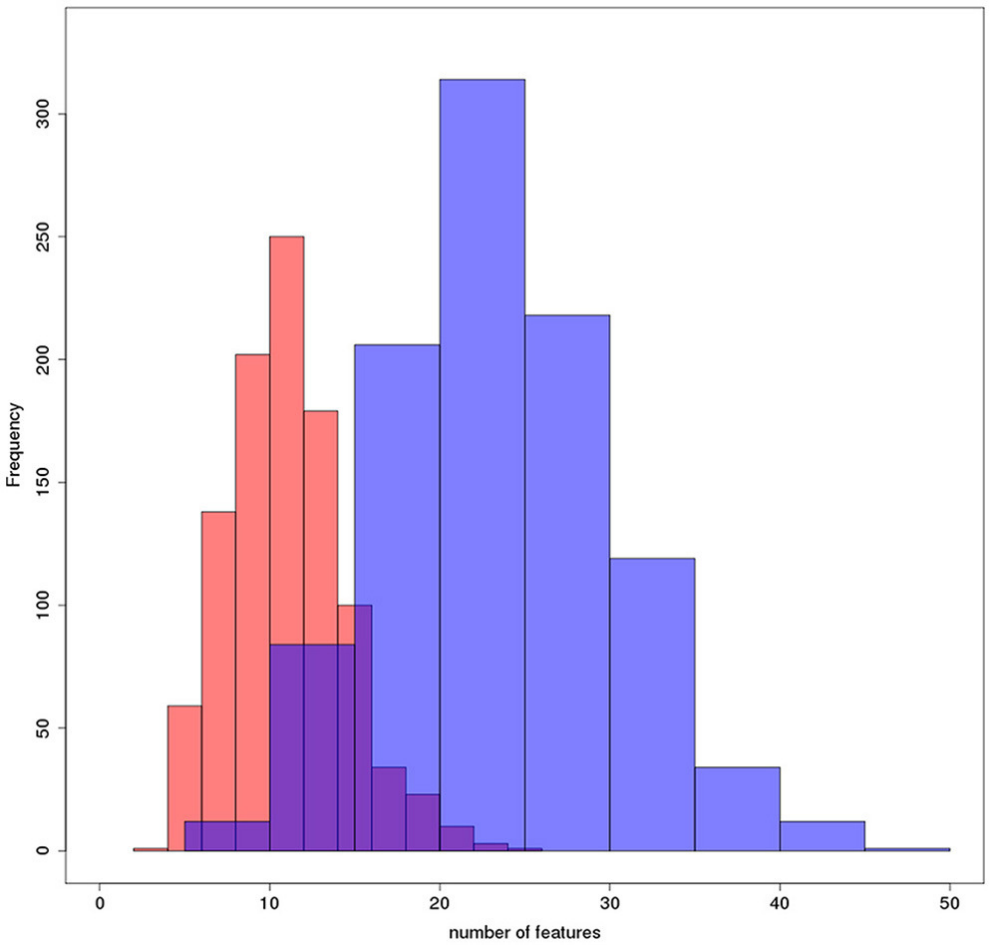
**Number of features selected per model**. Histograms plotting the frequency with which a number of features was selected across all bootstrapping iterations for elastic net (blue) and stepwise logistc regression (red).

The median VIF across all stepwise regression models and variables was 86, indicating a very high collinearity in the large majority of models. Mean area under the ROC curves in the test samples was 70% for the stepwise selection, and 80% for the elastic net regression models for the discrimination between AD cases and controls. The mean AUC and corresponding 2.5/97.5 percentile confidence intervals for both models are shown in Figure 2. The selected features are shown in Table 3 for both models, with seven features selected in at least 50% of 1000 cross-validation repetitions for the elastic net and two features selected for the stepwise logistic regression model. Figures 3, 4 show the frequency distribution of feature selection, suggesting that features were more homogeneously and more often selected in the cross-validation repetitions for the elastic net compared to the stepwise logistic regression models, with a median value of 10 features with the stepwise regression and 22 features in the elastic net regression.

**Table 3:** **Selected features**.

**Frequency [%]**	**Anatomical regions**	**Functional networks (Shirer et al., 2012)**
**FEATURES FROM ELASTIC NET**		
94.5	Left/right gyrus temporalis superior	Auditory network
87.1	Right gyrus frontalis superior <-> left gyrus occipitalis medialis	Basal ganglia network <-> visuospatial network
79.8	Left gyrus frontalis medialis <-> bilateral precuneus	Anterior salience network <-> precuneus network
69.9	Left/right precentral gyrus	Sensorimotor network
68.7	Right gyrus frontalis inferior <-> cingulate gyrus body	Anterior salience network <-> dorsal DMN
60.9	Right gyrus angularis <-> right gyrus frontalis medialis	Dorsal DMN <-> right executive control network
59.9	Bilateral anterior cingulate gyrus/ left gyrus frontalis superior/left gyrus frontalis medialis <-> left lobulus parietalis inferior/superior	Dorsal DMN <-> left executive control network
**FEATURES FROM STEPWISE LOGISTIC REGRESSION**		
56.9	Left/right precentral gyrus	Sensorimotor network
53.8	Right gyrus frontalis superior <-> left gyrus occipitalis medialis	Basal ganglia network <-> visuospatial network

When we repeated the analyses with dummy coded center covariates forced into the models, AUC was 81% for the elastic net penalty, and selected features above 50% frequency were unchanged. For the stepwise regression, AUC decreased to 68%, and no feature was selected with a frequency above 45%.

## Discussion

In accordance with our hypothesis, we found more accurate group discrimination between AD dementia cases and controls and more homogeneous feature selection from resting state fMRI data when using regularized logistic regression with an elastic net penalty compared with a classical stepwise logistic regression. These findings support the notion that regularized regression is superior to classical stepwise feature selection for dealing with highly collinear multidimensional functional imaging data. The features retrieved from penalized regression point to alterations of an extended functional network in mild AD dementia, compromising the dorsal DMN, but also key regions for language processing, object recognition and attention.

As illustrated by the high VIF with a median value of 86 (values above five are considered indicative for serious multicollinearity; Belsley, 1991), the regional rs-fMRI values exhibited a high degree of collinearity that compromised unbiased feature selection and determining the relevance of single features. The problem of dealing with multidimensional, multicollinear data is well-known in the statistical literature under the term of “the curse of dimensionality” (Bellman, 1961). Penalized regression has been developed since the 1940s to deal with this problem, encompassing techniques like ridge regression (Hoerl, 1970), the Lasso (Tibshirani, 1996), and more recently elastic net regression (Zou and Hastie, 2005), which combines both regularization techniques within the same model. Different to ridge regression, and similar to the Lasso, elastic net regression not only shrinks the feature coefficients but sets some of the coefficients to zero, thus reducing the dimensionality of the feature space. Different to the Lasso, elastic net regression is designed to select highly correlated features as a group rather than selecting only a single feature out of such a set of highly correlated variables, thus preserving a potentially meaningful correlation structure of the original feature space (Zou and Hastie, 2005).

Previous neuroimaging studies have successfully applied elastic net regression to multimodal neuroimaging data for feature selection for dementia prediction in subjects with mild cognitive impairment (MCI), and AD cases (Trzepacz et al., 2014; Teipel S. J. et al., 2015; de Vos et al., 2016). A previous study has applied this approach to rs-fMRI data of people with mild to moderate AD dementia from one scanner (Schouten et al., 2016), reaching 77% accuracy in the mild AD subgroup. In our multicenter study, cross-validated accuracy of 80% discrimination between AD cases and controls from elastic net regression was higher than the accuracy in this previous study (Schouten et al., 2016), but still lower than results from previous monocenter studies lacking cross-validation (Koch et al., 2012; Balthazar et al., 2014). Our findings level of accurcy agrees with estimates from previous cross-validated monocenter studies using non-linear machine learning techniques for classification (Challis et al., 2015; Dyrba et al., 2015). One recent study yielded 100% group discrimination between 20 AD patients and 20 controls using support vector machine classification (Khazaee et al., 2015). From the method description, however, the feature selection prior to cross-validated machine learning was based on the entire data set and was not part of the cross-validation.

Features selected by the elastic net regression were more consistent across repeated cross-validations than features selected by stepwise regression. Previous research on rs-fMRI in AD dementia has often focused on the DMN regions (Greicius et al., 2004; Koch et al., 2012; Balthazar et al., 2014). This approach reduces potential problems from collinearity through a priori feature selection. At the same time, it restricts the analysis to a single preselected functional network. Using elastic net regression, we retrieved the dorsal part of the DMN as key part of altered functional connectivity in AD. This agrees with previous analyses based on preselected DMN regions (Greicius et al., 2004; Koch et al., 2012; Balthazar et al., 2014) and underscores the overall validity of our approach. In addition, we found decreased functional connectivity in AD in the superior temporal gyrus, a region that is involved in language processing (Zhuang et al., 2014), and prefrontal parts of the salience network, prefrontal and parietal components of executive control networks, as well as the medial occipital gyrus as part of the ventral visual stream involved in object recognition (Teipel et al., 2007) and recognition of limb movements (Astafiev et al., 2004). These findings support the extended nature of AD pathology affecting several higher order cognitive networks, as previously found in topographic lesion driven studies (Grothe et al., 2016) and rs-fMRI analysis in small samples of 12 to 16 AD cases and 12–22 controls (Zhou et al., 2010; Agosta et al., 2012; Dai et al., 2012), and one large scale study (Brier et al., 2012). Different to two of these previous studies (Zhou et al., 2010; Agosta et al., 2012), we found only reductions, but no increases of functional connectivity in AD. This difference may have two possible causes. The first possible cause would be different severity of disease within the dementia stage of AD. However, the MMSE scores were similar between the AD cases of our and the previous studies. Another cause may be the different metric used as prediction features: we used correlation between regions irrespective of preselected networks, whereas the previous studies used regional loadings on independent components associated with specific functional networks (Agosta et al., 2012; Zhou et al., 2010).

Compared with elastic net regression, stepwise regression yielded only 70% accuracy. In addition, selection of the most relevant features was much less consistent across the 1000 iterations, compromising only two functional connections between sensorimotor and visuospatial regions, and no connection involving the DMN. These findings suggest that feature selection in step-wise regression was more sensitive to multicollinearity, where small differences in explained variance drive almost arbitrarily selection of a single feature among a set of highly collinear variables (Farrar and Glauber, 1967).

Stepwise logistic regression was sensitive to scanner effects, with a slight drop in prediction accuracy and a further loss of consistency in feature selection when scanner was forced into the model. In contrast, elastic net regression was insensitive to scanner effects; both accuracy of group discrimination and frequency of feature selection were unaffected when we repeated the analyses with scanner forced into the cross-validated models. This finding is of particular relevance given the sensitivity of rs-fMRI data to multiscanner effects, as has been reported in test-retest studies of rs-fMRI even in healthy people repeatedly scanned at the same scanner (Meindl et al., 2010; Chen et al., 2015; Lin et al., 2015; Orban et al., 2015; Shirer et al., 2015; Jovicich et al., 2016), including long-term evaluation after more than 12 months (Chou et al., 2012; Guo et al., 2012; Blautzik et al., 2013). Moreover, the use of multiple scanners typically results in high variability of signal-to-noise and contrast-to-noise ratios, particularly when using field strengths of 3T and higher (Magnotta et al., 2006; Lin et al., 2015; Jovicich et al., 2016).

We need to consider two main limitations of our study. First, the scan protocols were different between scanners. Multiscanner acquisition helps to increase sample size, a problem of many previous monocenter studies. In addition, estimates of accuracy derived from multicenter data may more easily generalize to future use of an imaging technology in routine care than estimates derived from single center data acquisition. We employed preprocessing steps that had been shown in a previous study to reduce multiscanner effects (Shirer et al., 2015), and used cross-correlation of regional signal time series which in a previous study had yielded more stable results across scanners than other connectivity metrics, such as cross-coherence or partial cross-correlation (Fiecas et al., 2013). Secondly, the reference standard in our sample was a clinical diagnosis of AD dementia, but independent PET or CSF based biomarker validation was not available in the majority of cases. Data came from expert centers experienced in the early diagnosis of AD. Still, a final judgment of the added value of rs-fMRI for AD diagnosis must await systematic evaluation of diagnostic accuracy in multicenter data from biomarker stratified cases.

In summary our findings point to an extended network of functional disconnection, including the dorsal DMN, but also involving functional networks employed in attention, object recognition and language processing. In a multicenter sample of AD and control cases, elastic net regression yielded cross-validated diagnostic accuracy that approached, but did not reach, the benchmark for a useful biomarker of AD (Consensus-Group, 1998); diagnostic approaches based on stepwise regression came not even close to this benchmark. These findings question the future wide-spread use of rs-fMRI as a stand-alone diagnostic marker of AD (Teipel S. et al., 2015). This does not exclude an important role of rs-fMRI as add-on diagnostic marker (Dai et al., 2012) and to identify mechanisms of functional disconnection and resilience in future prospective studies. Our data suggest that regularized regression should be preferred over still more widely used but less robust stepwise feature selection to retrieve homogeneous and stable estimates of altered functional networks in AD.

## Ethics statement

Institutional Review Board of the University Medicine Rostock. Written informed consent was provided by all subjects, or their representatives. The study was approved by local ethics committees at each of the participating centers, and has been conducted in accord with the Helsinki Declaration of 1975. For people with dementia, informed consent involves oral and written presentation of study procedures, and information of caregivers.

## Author contributions

ST: Conception of the work; acquisition, analysis, and interpretation of data for the work; drafting the work; final approval of the version to be published; agreement to be accountable for all aspects of the work in ensuring that questions related to the accuracy or integrity of any part of the work are appropriately investigated and resolved. MG, MD: Acquisition, analysis, and interpretation of data for the work; revising the work critically for important intellectual content; final approval of the version to be published; agreement to be accountable for all aspects of the work in ensuring that questions related to the accuracy or integrity of any part of the work are appropriately investigated and resolved. CM, TG, CS, ME, NF, EM, SK, VB, MW: Interpretation of data for the work; revising the work critically for important intellectual content; final approval of the version to be published; agreement to be accountable for all aspects of the work in ensuring that questions related to the accuracy or integrity of any part of the work are appropriately investigated and resolved.

### Conflict of interest statement

The authors declare that the research was conducted in the absence of any commercial or financial relationships that could be construed as a potential conflict of interest.
