# Supplementary material for: Robust Detection of Impaired Resting State Functional Connectivity Networks in Alzheimer's Disease Using Elastic Net Regularized Regression
Source: Front Aging Neurosci. 2017 Jan 4;8:318. doi: 10.3389/fnagi.2016.00318 (PMC5209379; doi:10.3389/fnagi.2016.00318)
Supplement: Supplementary file 1 [file DataSheet1.pdf]

Appendix: Penalized regression

The following description except the elastic net penalty follows the description of penalized regression in James et al. (1, Chapter 6.2).

To address the curse of dimensionality (2), the parameter estimate β from the classical linear regression model

$$\mathrm{RSS} = \sum_{i=1}^{n}\left(y_i - \beta_0 - \sum_{j=1}^{p}\beta_j x_{ij}\right)^2,$$

with the number of cases n and number of features p, has been constrained by various penalty terms with different ensuing characteristics of the estimates of the β coefficients.

The penalty terms decrease fit variance at the cost of increasing bias. Through the *variance-bias trade-off*, overall fit of a penalized parameter estimate can be improved over the simple least square estimation of the parameter without penalization.

In 1970, Hoerl and Kennard (3) introduced the **ridge penalty**, with an L2 quadratic penalty

$$\sum_{i=1}^{n}\left(y_i - \beta_0 - \sum_{j=1}^{p}\beta_j x_{ij}\right)^2 + \lambda\sum_{j=1}^{p}\beta_j^2 = \mathrm{RSS} + \lambda\sum_{j=1}^{p}\beta_j^2,$$

with the tuning parameter $\lambda \geq 0$, and the shrinkage penalty $\lambda\sum_j \beta_j^2$, and the number of features p. This penalty shrinks the magnitude of all coefficients, but sets no coefficient exactly to zero. As a result, it increases prediction accuracy, but has limited model interpretability in the presence of a high number of features.

In 1996, Tibshirani (4) described the **Least Absolute Shrinkage and Selection Operator (Lasso)**, which is characterized by an L1 (absolute value) penalty,

$$\sum_{i=1}^{n}\left(y_i - \beta_0 - \sum_{j=1}^{p}\beta_j x_{ij}\right)^2 + \lambda\sum_{j=1}^{p}\left|\beta_j\right| = \mathrm{RSS} + \lambda\sum_{j=1}^{p}\left|\beta_j\right|,$$

with the tuning parameter $\lambda \geq 0$, the shrinkage penalty $\lambda\sum_j\left|\beta_j\right|$, and number of features p.

The Lasso forces some of the coefficients to become zero, so that it provides both shrinkage and subset selection. The fit for the Ridge regression is superior to the Lasso if many variables are truly related to the outcome, whereas the fit of the Lasso is superior to the Ridge if only few variables are truly related to the outcome.

For collinear data, which are characterized by subgroups of features with high intercorrelation, such as has to be expected in brain imaging data with hubs that are interconnected in partly overlapping networks, the Lasso has the unfavorable characteristics that it selects only one among a group of highly correlated features. In addition, if $p > n$, i.e. the number of features is larger than the number of cases, the Lasso selects at most n variables.

To overcome these limitations, in 2005, Zou and Hastie (5) introduced the **elastic net penalty** that was extended to non-linear regression, such as logistic regression, in 2010 (6). Elastic net penalty regression features both, the L2 norm (quadratic) Ridge and the L1 norm Lasso penalty that are governed by an additional parameter $\alpha\epsilon[0,1]$,

$$\sum_{i=1}^{n}\left(y_i - \beta_0 - \sum_{j=1}^{p}\beta_j x_{ij}\right)^2 + \lambda\sum_{j=1}^{p}\left[(1-\alpha)\frac{1}{2}\beta_j^2 + \alpha\left|\beta_j\right|\right]$$
$$= \text{RSS} + \lambda\sum_{j=1}^{p}\left[(1-\alpha)\frac{1}{2}\beta_j^2 + \alpha\left|\beta_j\right|\right].$$

If $\alpha = 0$, the elastic net penalty becomes the Ridge penalty, while if $\alpha = 1$, the elastic net penalty becomes the Lasso penalty. In addition, in simulated data, the elastic net penalty has been shown to select or discard highly correlated features as a group (5).

References

1. James G, Witten D, Hastie T, Tibshirani R. *An Introduction to Statistical Learning*. New York, NY: Springer New York (2013).
2. Bellman R, Bellman RE. *Adaptive Control Processes: A Guided Tour*: Princeton University Press (1961). 255 p.
3. Hoerl AE, Kennard RW. Ridge Regression: Biased Estimation for Nonorthogonal Problems. *Technometrics* (1970) **12**(1):55-67. doi: 10.1080/00401706.1970.10488634.
4. Tibshirani R. Regression Shrinkage and Selection Via the Lasso. *Journal of the Royal Statistical Society, Series B* (1996) **58**(1):267–88.
5. Zou H, Hastie T. Regularization and variable selection via the elastic net. *Journal of the Royal Statistical Society: Series B (Statistical Methodology)* (2005) **67**(2):301-20. doi: 10.1111/j.1467-9868.2005.00503.x.
6. Friedman J, Hastie T, Tibshirani R. Regularization Paths for Generalized Linear Models via Coordinate Descent. *Journal of statistical software* (2010) **33**(1):1-22. PubMed PMID: 20808728; PubMed Central PMCID: PMC2929880.
